# Identifying Key Node with Motif-Based PageRank on Acupoint-Disease Network

**DOI:** 10.1155/2023/6101751

**Published:** 2023-12-04

**Authors:** Xuelong Yu, Xiao Liu, Li Luo, Hai Zhao

**Affiliations:** ^1^Engineering Research Center of Security Technology of Complex Network System, School of Computer Science and Engineering, Northeastern University, Shenyang 110169, China; ^2^Beijing PERCENT Technology Group Co. Ltd., Beijing 100096, China; ^3^School of Computer Communication, Lanzhou University of Technology, Lanzhou 730050, China

## Abstract

Existing research combines acupuncture theory with network science and proposes a new paradigm for the study of acupoint selection patterns—a key acupoint mining algorithm based on acupoint networks. However, the basic idea of this study for finding key acupoints is based on binary acupoint synergy relationships, which ignores the higher-order synergy among multiple acupoints and does not truly reflect the implicit patterns of each acupoint among meridian systems. Moreover, the mining results assessment method, which this new paradigm involves, does not have wide applicability and universality. In this paper, with the introduction of higher-order interactions between multiple acupoints, a high-specificity key acupoint mining algorithm based on 3-node motif is proposed in the acupoint-disease network (ADN). In response to the narrow applicability of the new research paradigm involving the evaluation of algorithms' measures, new and widely applicable and universal evaluation criteria are introduced in terms of resolution, network loss, and accuracy, respectively. Based on the principles of acupoint selection involved in acupuncture clinics in Chinese medicine, the acupoints involved in the data were divided into a total of 19 regions according to their distribution characteristics. From these 19 regions, we selected the key acupoints that have a large impact on the global network. Finally, we compared this algorithm with five other acupoint importance assessment algorithms in terms of resolution, network loss, and accuracy, respectively. The comprehensive results show that the algorithm identifies key acupoints with an accuracy of 63%, which is 14% to 21% higher than other existing methods. The key acupoints identified by the algorithm have a significant disruptive effect on the connectivity of the network, indicating that the key acupoints are at the core of the acupoint-disease network topology. They have a significant propagation influence on other acupoints, which means that the key acupoints have high-synergistic cooperation with other acupoints. Meanwhile, the stability and specificity of the algorithm ensure the reliability of the key acupoints. We believe that the key acupoints identified by the algorithm can be used as core acupoints from the perspective of network topology and high synergy of other acupoints, respectively, and help researchers explore targeted and high-impact combinations of acupoints to optimize existing acupuncture prescriptions under condition constraints.

## 1. Introduction

As a traditional medical method, acupuncture has important medical value and reliable clinical efficacy and has been recognized by many international organizations [1]. In traditional Chinese medicine (TCM), acupuncture is a method in which the TCM doctor selects the appropriate acupoints along the affected meridians for the disease and solves the clinical problem through needling, pressure, or thermal stimulation. With the rapid development of modern medicine, a large number of scholars have launched an exploration of the potential medical value of the meridian system, the mechanisms of interaction with other organs, and the full range of information about acupoints [2–5]. In addition, researchers have summarized the common acupoints and selection patterns for specific diseases based on historical treatment case data [6–9]. Scholars in other fields have also studied the characteristics of acupoint selection for treatment of diseases using the bioelectric potential of acupoints and their standard errors [10].

The human meridian system is a network of interwoven meridians and collaterals. It forms a complex structural and functional system along with other systems of the body. Meridians are scattered throughout the body and interact with other subsystems to transmit and regulate the physiological information of the body. This coincides with the idea of network science. The use of complex network thinking to explore the laws of acupoint selection has become a new trend in TCM research, where the identification of key nodes in complex network analysis methods can support new insights into the study of acupoint-disease relationships. There has been much literature on the preliminary application of complex network theory to the study of acupoint-disease relationships [11, 12]. On this basis, there is also the use of complex networks to study the combination pattern of acupuncture points for different diseases [13–18]. The characteristics of the above literature are shown in Table 1.

However, most of the aforementioned literature uses network-based statistical analysis to study combinations of acupoints, but has not yet gone further to use network thinking to analyze the relationship between acupoints and diseases and to dig deeper into the information contained in the network. Shi et al. [19] investigated a new method of supplementary acupoint selection with in-depth reference to complex network theory. This research work constructed a weighted undirected acupoint-disease network (ADN) based on clinical acupuncture prescription literature. Based on ADN, the key node identification method is used and three evaluation metrics (resolution, network loss, and accuracy) are introduced to select key points for each meridian. Among them, resolution measures the ability of the algorithm to distinguish the size of the effect produced by the acupoints in a specific region or at a specific range. Network loss measures the ability of the key acupoints identified by the algorithm to transfer physiological information between the target regions. Accuracy measures the ability of the key acupoints mined by the algorithm to produce a synergistic size with other acupoints. Under these three metrics, higher scores indicate that the algorithm is stronger in the corresponding ability and vice versa.

From the perspective of clinical acupuncture in TCM, this new research idea has more far-reaching implications. First, exploring key acupoints from ADN can be understood as analyzing the abstract distance between acupoints from a large amount of clinical prescription data. Second, it helps to understand the macroscopic connections between acupoints from the perspective of symptoms.

Networks proposed in the aforementioned literature are obtained based on the idea of constructing binary relationships that express the interactions between nodes in their most simplified form. For example, the network was constructed from clinical acupuncture prescription data based on the principle that two acupoints act on at least one disease. However, the binary relationship ignores more information about multiple interactions.

That is, the key nodes obtained from the above literature are not synergistic with other nodes and have a strong isolation, which may be ineffective if such acupoints are used in conjunction with other acupoints to treat a disease. In clinical acupuncture prescriptions, multiple acupoints act simultaneously on a disease, i.e., multiple acupoints produce higher-order interactions. This higher-order interaction indicates that multiple acupoints simultaneously regulate physiological information in the body. Obviously, such higher-order interactions cannot be ignored. Accordingly, key acupoint mining algorithms that ignore such higher-order interactions can lead to inaccurate key acupoints obtained. The higher-order interactions of multiple acupoints can reflect not only the cooperative ability of multiple acupoints but also the potential connections between multiple acupoints. Mining higher-order interactions between multiple acupoints can lead to potential combinations of acupoints with high-synergistic ability. At the same time, the resolution, network loss, and accuracy introduced in [19] have a narrow applicability and are not able to evaluate and reflect the higher-order interaction ability between nodes.

In this paper, based on the above description, the motif-based PageRank (MPR) algorithm is proposed based on the research work of [19]. The algorithm is based on the ADN, which reconstructs the weights of the network edges by measuring the amount of synergistic information in each triplet to obtain a new synergy matrix. Using the background knowledge of Chinese medicine, the ADN is divided into 19 regions. Then, the PageRank algorithm was used to find the key acupoints for each region. In brief, the contributions of this work are summarized as follows:

- In response to the binary relationship that would ignore the multivariate interaction information, a higher-order interaction of multiple acupoints is introduced in the ADN network, and the interaction is expressed in the form of a 3-node modulus.
- Due to the introduction of higher-order interactions, the conventional adjacency weight matrix cannot express higher-order interactions. Therefore, we reconstructed the adjacency weight matrix of the binary relationship network to obtain a synergistic strength matrix containing multiple synergistic relationships. Then, the PageRank algorithm was used to find the key points for each region.
- The network loss and accuracy calculation methods introduced in [19] cannot measure the goodness of key acupoint mining algorithms with higher-order interactions, so we introduce new and widely applicable calculation methods.

## 2. Materials and Methods

### 2.1. Data Availability

The data source used in this paper consists of two parts. The first part was selected from the 1994 acupuncture prescriptions for 50 common conditions used in [19, 20], which is a summary of the acupuncture point selection for 50 common conditions recorded in 5733 Chinese acupuncture clinical literature. To indicate that the key point mining algorithm is not dependent on a specific dataset, the most recent acupuncture literature data was chosen for the second part. The changes of major acupoints in ancient and modern acupuncture treatments over time were explored in [21], which involved modern acupuncture treatment literature from the CDSR (Cochrane Database of Systematic Reviews) with high authority. Therefore, we chose CDSR as the primary source of data for the modern acupuncture treatment literature.

### 2.2. Data Cleaning and Extraction

**Figure 1:**
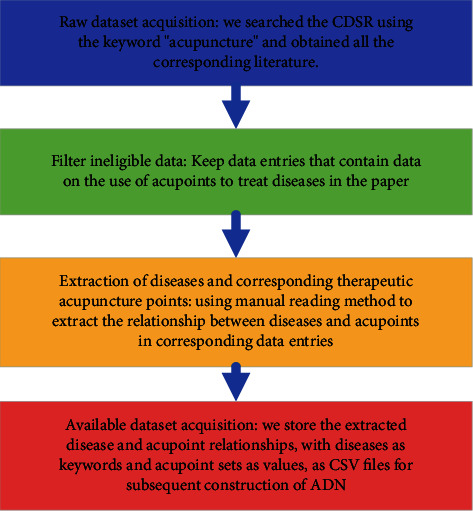
Data acquisition process for the second part of the dataset.

The first part of the dataset records the relationship between the disease and the corresponding acupoint set, so it can be collected directly using key-value pairs, where the disease is the keyword and the acupoint set is the corresponding value. For the second part of the dataset, the process described in Figure 1 is used for collection, as shown in Figure 1.

Since these two datasets cannot guarantee the unbiasedness of the dataset itself, in order to be able to include more special cases and considering more cases, we merged these two datasets into one dataset to provide data support for the subsequent work.

### 2.3. Acupoint-Disease Network (ADN)

The key-value pairs in the data formally describe the synergistic therapeutic effect of multiple acupoints on the disease, which implies the association relationship between the acupoints. In this paper, we use the ADN network proposed in [19], which is an acupoint association network obtained from the construction of acupoints based on the synergistic effect of the acupoint set on the disease. The formal representation of the network is specified as follows:

The key-value pairs in the data formally describe the synergistic therapeutic effect of multiple acupoints in the acupoint set on the disease, which implies the association relationship between the acupoints. In this paper, we used the ADN from [19], which uses acupoints as nodes and establishes a link between any two nodes when they appear in one disease at the same time, with the number of disease types that appear as the weight of that edge. The formal representation of the network is specified as follows:

**Definition 1 .**

*G* represents an undirected acupoint-disease network.

(1) $$\begin{array}{c} G=\left(V,E,W\left(E\right)\right)\text{,} \end{array}$$

where *V* denotes the set of acupoint nodes of the network, i.e., the set of acupoints. *E* denotes the set of edges of the network, i.e., the set of association relationships between acupoints. If two acupoints act on one disease at the same time, an association relationship is established between these two acupoints. If two acupoints act on more than one disease, the strength of the association between these two acupoints is expressed in the form of weights, where *W*(*E*) denotes the weight distribution of the association relationship between the acupoints.

**Figure 2:**
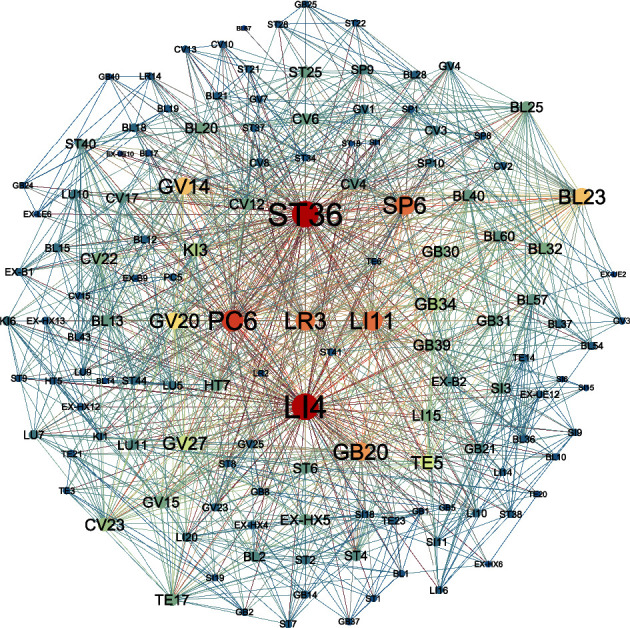
Network models of ADN: in the network model, the color and width of edge reflect different weights and the size of node reflects the frequency of utilization.

Figure 2 shows the ADN network constructed from the data in this paper. First, using the “NetworkX” package in the Python programming language, a link is established between any two acupuncture points when they act on a disease at the same time. The number of diseases that the connection acts on is used as the weight of the connection. Second, a graph file is generated using the Python programming language and visualized using Gephi software. We analyzed the ADN in terms of attributes such as network degree distribution, average degree, average weighted discard, and network clustering coefficient. The results of network analysis are shown in Table 2.

**Figure 3:**
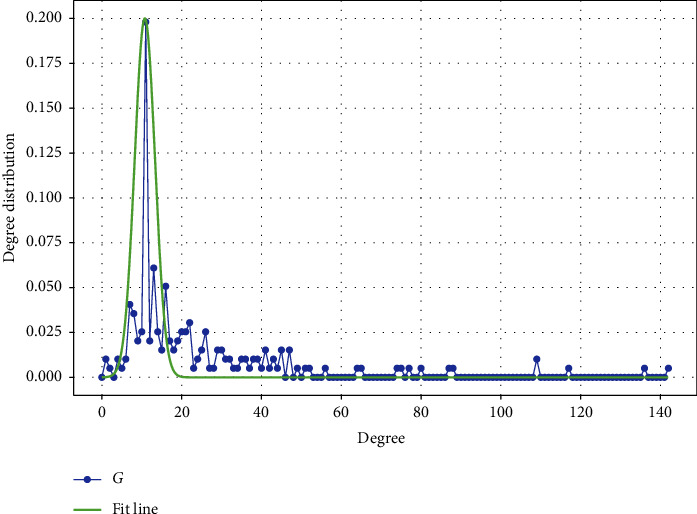
The degree distribution in ADN.

Figure 3 depicts the node degree distribution of the ADN. As can be seen in Figure 3, the degree distribution exhibits a small-world network characteristic, which is extremely close to the normal distribution (shown by the fitted line in the figure). The large number of node degrees in the network tends to be in the peak of the distribution curve, indicating that the node degrees in the network are not widely disparate. Similarly, this phenomenon can be seen in the properties of the network, as shown in Table 2.

The average clustering coefficient of the network describes the degree of close association between acupoints, and the average weighted degree describes the frequency of use of each acupoint. As seen in Table 2, the ADN has a high average clustering coefficient and average weighting, indicating that the acupoints are closely related to each other and that each acupoint is used more frequently. That is, the algorithm cannot distinguish the key acupoints on each meridian by these attributes alone, and it cannot help TCM doctors to find the best combination of acupoints for a specific disease with limited resources.

### 2.4. Problem Formulation

#### 2.4.1. Motif Definition

Since multiple acupoints produce synergistic effects in treating diseases, such synergistic effects cannot be expressed completely by pairwise relationships only, which will produce loss of information. Therefore, previous key acupoint mining algorithms established based on pairwise relationships loses the accuracy of key acupoints selected on each meridian. We used theoretically mature network motifs in complex networks for the description of multipoint synergy, on the basis of which the corresponding key acupoint mining algorithm was investigated. Since the description of multiacupoint synergy is too complex, we use 3-node motifs for the description in order to facilitate the abstraction of such a relationship [22].

**Definition 2 .**

*M* denotes the motif defined on *k* nodes. A motif *M* is defined on *k* nodes by a tuple (*B*, *A*), where *B* is a *k* × *k* binary matrix, and *A* ⊂ {1,2,…, *k*} specifies the anchor set, which is the set of the indices of the anchor nodes.

According to Definition 2, we give a toy model to explain the meaning of the motif.

**Figure 4:**
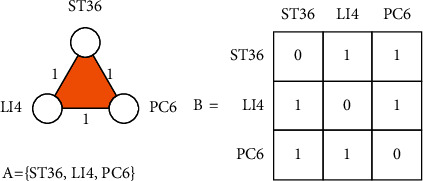
The example of 3-node simple motif.

An example of a 3-node simple motif is given in Figure 4. The weighted edges connected between nodes indicate multiple simultaneous occurrences of two nodes in the disease treatment prescription, where 1 indicates the number of occurrences and 0 indicates that there are no edges between the nodes. The orange surface indicates the synergistic relationship of these three nodes, and the geometric area of this triangle indicates the potential synergistic strength of these three nodes. The *B* matrix in Figure 4 is the mathematical form representation of the corresponding motif, and *A* is the node anchor set.

#### 2.4.2. Potential Synergistic Strength Distribution

**Figure 5:**
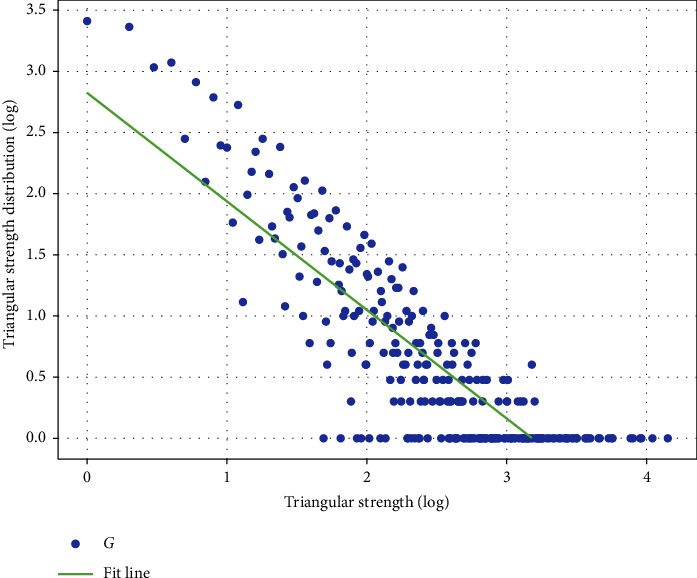
Potential synergistic strength distribution.

We extracted all the 3-node motifs in the ADN and calculated the distribution of synergy strengths of these motifs, and the results show that the synergy strengths of 3-node motifs in the network have the characteristics of power-law distribution, which means that these synergy strengths are significantly different. To characterize the power-law distribution, we use a double logarithmic coordinate system to demonstrate the potential synergy distribution of the network, as shown in Figure 5.

In Figure 5, the horizontal coordinate indicates the magnitude of synergy strength and the vertical coordinate indicates the probability index of synergy strength. As can be seen, the smaller the strength of the acupoint synergy, the greater the probability of its occurrence in the network and vice versa. With such a clear synergistic relationship of strength, the key acupoints on the meridians can be identified more accurately. The algorithm of key acupoint node mining based on synergistic relationship is given.

### 2.5. Key Acupoint Node Mining Based on Synergistic Relationship

A key node is a special node that plays an important role in a network. Since the propagation, synchronization, and control of physiological information by acupoints cannot be determined from the perspective of modern medicine, the highly abstract complex network model provides us with an effective basis for exploring key acupoints by describing the linking relationships among acupoints. In this work, we combine the ADN network with the actual fourteen meridian distributions and odd point distribution features and propose a key point mining algorithm that satisfies the fourteen meridian distributions and extrameridian point distribution features. In this paper, we continue to follow the definition of key acupoints in the literature [19], i.e., key acupoints are the acupoint indicated as the core has a wide spatial distribution of indications in acupoint network or the prescription. In a general way, the key acupoint mining algorithm consists of three main steps. First, acupoint clusters are divided according to meridian distribution features. Second, based on the ADN, the characteristics of the nodes in each cluster are extracted and then the importance of the nodes is evaluated. Finally, according to the key node evaluation index, the top 3 key acupoints nodes in each cluster are selected. Based on these steps, we designed a new node evaluation algorithm, the MPR algorithm, and selected a more convincing algorithm performance evaluation index.

#### 2.5.1. Acupoint Communities in the Meridian System

Due to the “selection of acupoints along the affected meridian” principle, different acupoints on the same meridian have a wide range of functions; more obviously, acupoints on certain meridians have the ability to modulate multiple symptoms locally. This makes it easy for the algorithm to focus the key acupoints on certain characteristic meridians. Therefore, we divide these acupoints based on the meridian system so that important acupoints with specific therapeutic effects can be highlighted in the network model and also balance the contribution of each community node to the network, helping TCM doctors to always have key acupoints as references in each target meridian.

There are 14 major meridians in the human body, which are the Shaoyin Heart Channel of Hand, the Taiyang Small Intestine Channel of Hand, the Taiyang Bladder Channel of Foot, the Shaoyin Kidney Channel of Foot, the Jueyin Pericardium Channel of Hand, the Shaoyang Sanjiao Channel of Hand, the Shaoyang Gallbladder Channel of Foot, the Jueyin Liver Channel of Foot, the Governing Vessel, and the Conception Vessel. Most of the body's acupoints are located on these 14 meridians. In addition to these acupoints, many extrameridian points are scattered on the surface of the body. The TCM doctor selects the targeted extrameridian acupoints according to the principles of proximal and symptomatic selection of acupuncture points in the area where the disease occurs. In Chinese medicine, extrameridian acupoints are classified as Points of Head and Neck, Points of Chest and Abdomen, Points of Back, Points of Upper Extremities, and Points of Lower Extremities. In summary, the nodes in the ADN network were divided into 19 communities, including 14 meridian communities and 5 extrameridian acupoints communities.

#### 2.5.2. Motif-Based PageRank (MPR) Algorithm

First, we calculated the synergistic strength measurement of three-node motifs. In order to find the synergistic strength of each three-node motif, we make use of the *effective information* (EI) [23], which is a network measure that quantifies the volume of path information between acupoints in a network, as well as how that volume is distributed. Now, we give an equation for calculating the synergy strength of a network *G* as shown in the following equation:

(2) $$\begin{array}{c} EI\left(G\right)=H\left(\langle W_{i}^{out}\rangle \right)-\langle H\left(W_{i}^{out}\right)\rangle \text{,} \end{array}$$

where $H\left(X\right)=-\underset{x}{\sum }P\left(x\right)\log\;P\left(x\right)$. The first is the entropy of the average out-weight vector in the network, 〈*W*_*i*_^out^〉, which captures how distributed synergistic out-weights between acupoints in the network are. The second is the average entropy of each acupoint's *W*_*i*_^out^.

Since the ADN contains all the information of the node, the synergistic strength of the 3-node motifs that needs to be measured can be measured by removing the motifs from the ADN. Therefore, the synergistic strength of the 3-node motifs is calculated as shown in equation (3).

**Definition 3 .**

EI(∆_*i*_) represents the synergistic strength of the *i*-th motifs.

(3) $$\begin{array}{c} EI\left({∆}_{i}\right)=EI\left(G\right)-EI\left(G_{i}^{\prime }\right)\text{,} \end{array}$$

where the ADN with motif ∆_*i*_ removed is denoted as *G*_*i*_′.

Second, the synergy strength matrix *H* is given. The synergistic strength of all 3-node motifs in the network is obtained by equation (3). To better portray the importance of nodes, we used the well-established PageRank algorithm to calculate the importance of nodes. However, the algorithm still does not include synergy beyond pairwise relationships and only uses the binary relationship adjacency weight matrix of the network. Since the multinode synergy effect changes the actual strength of interactions between pairs of nodes. Therefore, we can convert the synergy strength of the 3-node motifs between two nodes to obtain a synergy strength matrix with a completely new one, replacing the adjacency weight matrix of the original network. Since paired nodes will appear in different 3-node motifs, we define the synergy strength of paired nodes based on 3-node motifs as the weights of the edges between nodes, as shown in the following equation:

(4) $$\begin{array}{c} S\left(v_{i},v_{j}\right)=\underset{v_{i},v_{j}\in {∆}_{k}}{\sum }EI\left({∆}_{k}\right)\text{.} \end{array}$$

**Figure 6:**
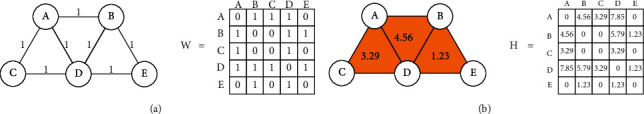
The example of weight matrix: (a) the traditional adjacency weight matrix; (b) the synergy strength matrix.

Figure 6 shows the pairwise relationship adjacency matrix compared with the synergistic strength matrix. The traditional adjacency weight matrix, as shown in Figure 6(a), can be regarded as the synergy strength matrix of paired nodes. For synergy with more than 2 nodes, the traditional adjacency weight matrix cannot portray such synergy, which will lose a lot of useful information, such that the accuracy of the measured node importance is not high.  Figure 6(b) shows the synergy strength matrix of the 3-node motifs, and the orange triangles are marked with the synergy strength of the corresponding motifs. Compared with the traditional adjacency weight matrix, the synergistic strength matrix obtained by equation (8) can more effectively utilize the synergistic information of multiple nodes to accurately capture the implied connections between nodes and accurately identify the importance of nodes. For example, the traditional adjacency weight matrix only captures the synergistic relationship between nodes *D* and *B*, while synergistic strength matrix based on the 3-node motifs can capture the synergistic relationship between nodes *A*, *B,* and *D*. Moreover, if the importance of nodes is discerned by their weighted degree (summing over the rows or columns of the matrix), the traditional adjacency weight matrix cannot distinguish the importance of nodes *A* and *B* and the importance of nodes *C* and *E*. In contrast, the importance of these five nodes can be accurately distinguished by the synergistic strength matrix shown in Figure 6(b).

Third, node importance is calculated by PageRank. PageRank was originally intended to calculate the importance of each web page under a given network formed by web pages in the Internet world and is widely used today [24]. However, in a general sense, PageRank reflects the reachability of other nodes to node *v*_*i*_ from a topological perspective by means of iterative computation. In [25], a simple iterative algorithm is used to compute the PageRank vector.

(5) $$\begin{array}{c} {\overset{⟶}{x}}_{t}=dP^{T}{\overset{⟶}{x}}_{t-1}+\frac{1-d}{N}\overset{⟶}{e}\text{,} \end{array}$$

where ${\overset{⟶}{x}}_{t}$ is the PageRank vector in step *t*, *x*_*i*_ is the PageRank value of the *i*-th node in *G*, and *N* is the number of nodes. $\overset{⟶}{e}\in R^{N}$ is a vector with every entry equal to 1. *P* is the transition probability matrix obtained by *P*_*ij*_=*W*_*ij*_/∑_*j*_*W*_*ij*_, where *W* is the weighted adjacency matrix of the graph and *W*_*ij*_ represents the weight of *e*_*ij*_. For a directed unweighted graph, *W*_*ij*_=1 if *e*_*ij*_ exists and *W*_*ij*_=0 otherwise. In [26], Bianchini et al. demonstrated that this iterative computation always converges, thus we obtain the PageRank value for each node in the graph.

In an unweighted undirected network, the more reachable paths to node *v*_*i*_, the larger its PR value; while in a weighted undirected network, the higher the weight of reachable paths to node *v*_*i*_, the larger its PR value. At the same time, from the perspective of synergy strength, the higher the PR value of a node, the higher the PR value of its neighbors will be influenced to some extent. Therefore, no matter original or weighted PageRank, the weights are calculated based on the direct relations between two nodes. In other words, they only consider the binary relations, while ignoring higher-order relations captured by motifs.

Given a network *G*, the PageRank value of each node represents influence or importance. *W*_*ij*_ represents the strength of relationship between node *v*_*i*_ and *v*_*j*_. From equation (5), we can see that *W* affects the transition probability matrix *P*, thus the final PageRank values. That is, by changing the matrix *W*, PageRank is able to capture the information hidden in the network. In summary, the adjacency matrix *H*, which contains multibody synergy, can provide more information than the matrix *W*, enabling PageRank to obtain the key nodes with potential maximum synergy strength with multiple nodes. Therefore, we replace the transfer probability matrix *P* in equation (5) with *P*_*H*_ (*P*_*ij*_=*H*_*ij*_/∑_*j*_*H*_*ij*_). The importance of nodes in the ADN network is calculated by the improved PageRank algorithm, which is called Motifs-based PageRank algorithm (MPR).

## 3. Results

### 3.1. Experimental Environments

The software used in this paper is shown in Table 3. Python, with its simplicity, ease of reading, and scalability, is the tool of choice for many researchers working with data. NetworkX is a package that is often used in Python for working with graph type data. Gephi is also commonly used by many researchers to visualize graphs.

### 3.2. Key Acupoint Node in Communities

In the analysis of the literature [19], it was shown that the disparity in the importance of the key acupoints calculated by the CC algorithm for each community is not significant, which indicates that the average distance from all nodes to other nodes is similar in the network topology. It further indicates that in addition to the truly important key acupoints that are selected when tuning local and global physiological information, many acupoints that are related but do not actually play an important role are also selected. This is actually due to the fact that the algorithm considers only the local binary relational topological information of the nodes, allowing all nodes to exhibit such similar results. The results of our proposed MPR algorithm compared with the CC algorithm for the selected key acupoints in each community are shown in Table 4. Among them, there are 14 communities with the same results and 5 communities with different results. Due to the limited amount of data, the number of nodes in some communities is less than 3. By comparing the results, our proposed algorithm makes it possible that the acupoints involved in the synergistic interaction of multiple nodes will be ranked higher, making them key acupoints and providing an accurate and valid reference object for TCM doctors.

### 3.3. Performance Evaluation

In order to verify the reliability of the key acupoints selected by MPR, three metrics, namely, resolution, network efficiency, and accuracy, will be used to evaluate the reliability of MPR, respectively. Meanwhile, a comparison experiment with six node importance evaluation metrics is conducted to verify the reliability of MPR. These importance evaluation metrics include closeness centrality (CC), betweenness centrality (BC), eigenvector centrality (EC), CLD algorithm [27], and key node algorithm [19]. The closeness centrality considers the reciprocal of the average shortest path distance to *u* over all *n *− 1 reachable nodes, which indicates that the higher the closeness centrality of *u*, the closer the other nodes are to *u*. Betweenness centrality considers the sum of the fraction of all-pairs shortest paths that pass through *u*, which indicates that the higher the betweenness centrality of *u*, the closer it is to the center of the network. The eigenvector centrality considers not only the degree of the node itself, but also the degree of the node's neighbors. The CLD integrates the local clustering characteristics of nodes and their neighbors, and is a new key node mining algorithm. The key node algorithm considers the product of weights on all paths from node *u* to other nodes, which measures the importance of the node in the global network.

#### 3.3.1. Resolution

Resolution is one of the common measures of algorithm performance and reflects the extent to which algorithms are able to distinguish acupoints with high similarity in the network [19]. We used the benchmark comparison algorithm with MPR to score the 197 acupoints involved in the data and compared the resolution of each algorithm. Resolution was calculated as shown in the following equation:

(6) $$\begin{array}{c} f\left(r_{A}\right)=1-\frac{\sum_{i=1}^{R}N_{i}^{2}}{R*N^{2}}\text{,} \end{array}$$

where *r*_*A*_ denotes the ranking result of the algorithm, *N* denotes the number of nodes in the network, *R* denotes the granularity of the ranking result, and *N*_*i*_ denotes the number of *i*-th nodes in the ranking result. When *R*=1, *f*(*r*_*A*_)=0 indicates that the ranking result cannot distinguish the importance of nodes in the network. The closer the *f*(*r*_*A*_) of the algorithm is to 1, the finer the granularity of the sorting result of the algorithm.

#### 3.3.2. Network Efficiency

We believe that key points, as important entry points for regulating local and systemic physiological states, can ensure a large, stable, and accurate transmission of physiological information between target regions, in other words, each key acupoint plays a “bridge” role in the overall network topology. Network efficiency measures the bridge role of nodes in the network in terms of shortest paths, i.e., the number of shortest paths through a node. The higher the number of shortest paths through a node, the greater the bridging role of the node, and vice versa. The network efficiency is calculated by removing the identified critical nodes of each algorithm and comparing the strengths and weaknesses of each algorithm in terms of network efficiency [28–30]. The network efficiency was calculated as shown in equation (7). The lower the network efficiency of the algorithm, the higher the central role of the key acupoints identified by the algorithm.

(7) $$\begin{array}{c} \eta =\frac{1}{N\left(N-1\right)}\underset{v_{i},v_{j}\in V,i\neq j}{\sum }d_{ij}\text{.} \end{array}$$

#### 3.3.3. Accuracy

In order to measure the accuracy of the key acupoints identified by the algorithm, a set of benchmark scores is needed. We chose a node capability assessment method for weighted networks, the weighted cascades (WC) model [31–33], to calculate the base synergistic capability of all nodes as a benchmark score. The accuracy of the set of key acupoints identified by each algorithm is evaluated by measuring the Kendall correlation coefficient between the benchmark scores and the node importance results calculated by each algorithm. The higher the correlation, the more accurate the node importance ranking results. The Kendall correlation coefficient is calculated as shown in the following equation:

(8) $$\begin{array}{c} \tau =\frac{2\left(X_{a}-X_{b}\right)}{\left|X\right|\left(\left|X\right|-1\right)}\text{.} \end{array}$$

Since key acupoints are able to regulate physiological information throughout the body and locally, they are able to transmit physiological information between different regions in a large, stable, and accurate manner, which coincides with the basic idea of the WC model. The WC model measures the synergistic ability of nodes with other nodes from the perspective of information diffusion. Because of its high applicability, the model has been widely applied by scholars as a benchmark method to measure the importance of nodes. In this model, the network is first converted into a directed network, and the diffusion probability between nodes is set by the calculation method shown in the following equation:

(9) $$\begin{array}{c} p_{ij}=\frac{W_{ij}}{\sum_{v_{k}\in N_{i}}W_{ik}}\text{.} \end{array}$$

The importance of each node is measured through the process of information dissemination on the ADN. To facilitate understanding, we use a toy example to demonstrate how this benchmark evaluation criteria work.

**Figure 7:**
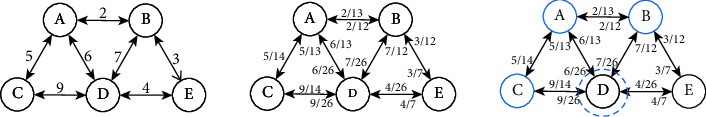
The toy examples for the WC model.

Figure 7 illustrates the computational process of the WC model. First, the network is transformed into a directed network (undirected edges and converted into bidirectional edges). Second, the weights of the edges of each node pointing to its neighbors are converted into propagation probabilities according to equation (9). Finally, on such a network, the WC propagation process is simulated. The number of other nodes that each node affects directly and indirectly is obtained as the importance of that node.

### 3.4. Experimental Results and Discussion

#### 3.4.1. Experimental Results

*(1) Resolution*. We compare the advantages and disadvantages of MPR and the five benchmark algorithms in terms of resolution by plotting the complementary cumulative distribution function (CCDF) curves.

**Figure 8:**
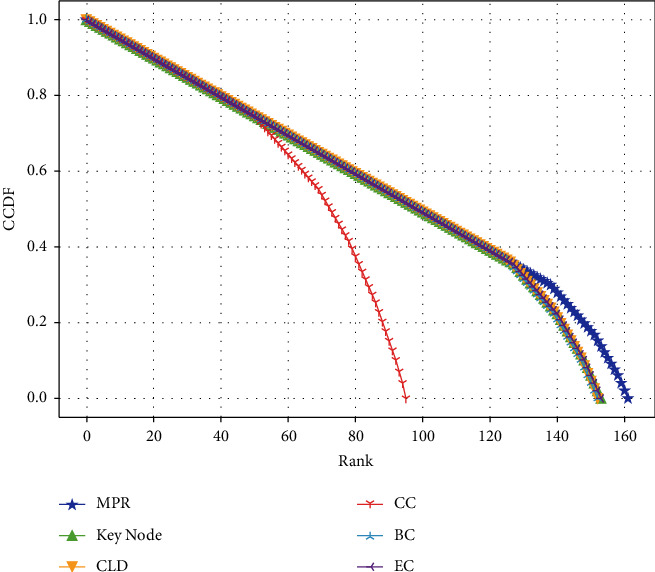
The complementary cumulative distribution function (CCDF) for the ranking distributions of ranking list offered by six algorithms.

As can be seen in Figure 8, the resolution of MPR is significantly better than the other five comparison algorithms, indicating that MPR has a more prominent advantage in resolution than the other five comparison algorithms. MPR utilizes the synergy of multiple nodes, which allows it to distinguish the synergistic ability of each acupuncture point involved in the data with other acupuncture points. In contrast, the other algorithms only use network topological information based on the pairwise relationships and have little ability to distinguish the synergy of acupoints with other acupoints, thus highlighting the importance of synergistic interactions of multiple nodes.

*(2) Network efficiency*. By calculating the network efficiency for each algorithm, the results are displayed in Table 5. From the results in Table 5, we can see that the worst network efficiency is the CC algorithm, while the best network efficiency is the MPR algorithm. From the perspective of network efficiency, the key acupoints identified by MPR play the role of “bridge” in the whole network topology.

*(3) Accuracy*. Since evaluating the results in this way is probabilistic, we repeated the propagation process 10 times and averaged the obtained results so that their experimental results do not have traces of artificial selection. We show the Kendall coefficients of each algorithm with respect to the benchmark results in Table 6. As can be seen in Table 6, the accuracy of the MPR algorithm far exceeds that of the other key point evaluation algorithms. This highlights the importance of the synergistic effect of multiple acupuncture points.

#### 3.4.2. Discussion

Network science, as a way of thinking that can explain potentially complex phenomena, provides a new perspective for understanding the role of acupoints in the meridian system in the association, transmission, control, and coordination of physiological information. To better explore the key acupoints in ADN, we improved our previous work [19]. We designed a new key acupoint mining algorithm based on the higher-order interactions of multiple nodes. The method far outperformed other key acupoint mining algorithms in the evaluation experiments with three different parameters. The MPR algorithm is able to mine not only the key acupoints on the fourteen meridians of the human body but also extrameridian points on different body parts. Using this feature, the TCM doctor can select the acupoints that have the greatest synergistic effect on the target meridians after diagnosis. Although the TCM doctor's acupuncture prescription may vary depending on previous experience, these key acupoints always play a central role. These points can be used as preferred acupoints for a variety of selection tools, providing a broader range of physiological modulation and a wider range of possible allocation patterns. The goal of our work is the same as that done previously [19], to accurately mark key acupoints to optimize and refine acupuncture prescriptions for TCM doctors for common symptoms.

Currently, network science has well-established research results. However, the networks underlying these theoretical results provide only a limited description of the real world, because such network models are constructed from pairwise interactions. In many biological, physical, and social systems, the basic elements of the network interact in groups, and such interactions do not always decompose into pairwise relational couplings. For example, evidence in neural systems suggests that higher-order influences are statistically and topologically present and important. Similarly, the acupoints chosen by the TCM doctor to treat a disease are synergistic at the same time. The concept of higher-order interactions is well known in many-body physics, such as strong interactions [34, 35] or van der Waals interactions [35], as well as statistical mechanics [36]. Several researches have shown that the presence of higher-order interactions can have a significant impact on the dynamics of networked systems, from diffusion [37, 38] and synchronization [39, 40] to social [41–43] and evolutionary processes [44].

A key acupoint mining algorithm based on higher-order interactions between acupoints can identify key acupoints with high-synergistic effects. In the human meridian system, the higher-order interactions between acupoints cannot be ignored. From the clinical perspective of acupuncture in Chinese medicine, a group of acupoints act together to regulate local and global physiological information for the purpose of healing. However, in previous studies of key acupoints, researchers' models were constructed based on pairwise relationships, ignoring higher-order interactions between acupoints. As a result, the models are not able to capture the more important and substantial information, making the accuracy and applicability of the results suffer considerably. In other words, higher-order interactions can reveal the functions and mechanisms of multiple acupoints in synergy, further explore new combinations of acupoints, and also reveal the robustness of the human meridian system. In general, a specific acupoint is selected for a specific disease and that acupoint has specific disease characteristics. However, it has been demonstrated in the literature [45] that the selection of an acupoint for a specific disease does not imply that the acupoint has a specific indication for that disease. In other words, there is not a one-to-one correspondence between disease and acupoint. The ADN network constructed by the disease-acupoint relationship implicitly expresses the relationship between acupoints at the representation level. Therefore, the key acupoints identified by the algorithm can help researchers to identify the best acupoint prescriptions faster and more accurately when analyzing local symptoms.

From the perspective of network, key acupoints have a central position in the network and deeply influence the network connectivity, acupoint synergy strength and network information diffusion. The key acupoint mining algorithm based on higher-order interactions captures not only the path information of the network but also the synergy information between multiple acupoints, which can accurately evaluate each acupoint its influence in the network and distinguish the importance of each acupoint. In addition, the MPR algorithm uses a new synergistic strength matrix based on the 3-node motifs, so that the edge weights of the network are expressed as the cumulative synergistic strength of the multiple modalities in which the edge is located. Based on this, the higher-order interactions of multiple acupoints allow the acupoints to obtain different weight gains of higher-order interactions as a way to increase their value in the network. Acupoints with higher weighting gains have greater disease regulation ability with more Acupoint combination patterns. That is, from the perspective of higher-order interactions of acupoints, key acupoint nodes can be used as core acupoints with other acupoints to modulate local physiological states and help researchers explore combinations of acupoints with high-synergistic effects.

From an empirical perspective, the key acupoints explored from ADN have a strong stability in the diagnostic pattern. The diversity of acupoints used in acupuncture treatment is a result of the diagnostic patterns of different practitioners. The choice of diagnostic modality depends on the clinical experience and medical knowledge of the physician, and bias in the outcome of modality selection is inevitable. In [46], the authors showed by using network and text mining analysis methods that the best acupoints for a specific disease can be determined by analyzing diagnostic patterns. Medical data extracted from case reports in [46] to reveal the association between such patterns and acupoints prescribed in clinical practice. In [46], the five most common diagnostic patterns are listed and the five highest frequencies of acupoints in the corresponding diagnostic pattern are given and calculated separately. The most frequent ones, ST36, LI4, KI3, LR3, and SP6, are included in the set of key acupoints identified by the algorithm. Thus, the key acupoints showed a strong stability in the diagnostic pattern. When selecting certain prescription acupoints based on symptoms, prioritizing key acupoints has implications for improving the accuracy of prescriptions.

Inevitably, this research has some limitations. The ADN network is still constructed based on pairwise relationships, and the difference from the previous work is that this paper's work introduces 3-node higher-order interactions to reconstruct the weights of the network. From the experimental results, the MPR algorithm outperforms other algorithms in terms of accuracy, resolution and network efficiency, and has better adaptability and accuracy. The key acupoint mining algorithm using higher-order interactions is able to capture more valid information in the network, which helps to explore the potential relationships between acupoints from the perspective of higher-order interactions. However, the information of disease acupoints contained in the network is still limited by the ability to represent pairwise relationships. To address the shortcomings of network representation, in addition to introducing the concept of modalities in the network, higher-order networks can maximize the representation of higher-order interactions among acupoints. Second, due to the limitation of experimental data, the current research on the existence and visibility of the human meridian system has made significant progress, but the full distribution information of human meridians is not available, which makes the data sources available with different degrees of information loss. In future research, we will use higher-order networks such as hypergraphs or simple complex to break through the limitations of the original networks and further investigate the key acupoint groups and the potential associations between acupoints. The synergy between acupoints makes the potential association information among acupoints, and the mining and prediction of this potential association information by relying on the higher-order link prediction method can help researchers to reveal the functional and distribution characteristics of human meridians.

## 4. Conclusions

As a traditional medical method, acupuncture has an important medical value and reliable clinical efficacy. Currently, scholars have applied network science initially to the study of disease-acupoint relationships. This research work constructs an ADN based on clinical acupuncture prescription literature. By introducing higher-order interactions of 3-node motifs on the ADN, the edge weights of the network are reconstructed, and the MPR key acupoint mining algorithm is proposed. Using the MPR algorithm, key acupoints are selected for each region. Compared with other evaluation metrics, the higher accuracy of this metric culture can provide a better combination of acupuncture points as a reference. On this basis, the acupuncture prescription can be optimized and improved, which will reduce the differences in acupoint selection caused by subjective factors and improve the efficiency and effectiveness of acupuncture treatment. In future studies, we will use higher-order networks such as hypergraphs or simple complex to break through the limitations of the original network and further investigate the potential associations between acupoints. Second, for specific symptoms, we collect fine-grained data corresponding to the symptoms and model them in a more refined manner to find key combinations of acupoints for specific symptoms.

## Figures and Tables

**Table 1:** Literature review summary.

Paper	Experimental categories	Network building	Analysis method	
			Statistical analysis	Network analysis
[6]	Traditional controlled experiments		✓	
[7]	Traditional controlled experiments		✓	
[8]	Traditional controlled experiments		✓	
[9]	Historical case analysis		✓	
[12]	Analysis of complex networks	✓	✓	
[13]	Analysis of complex networks	✓	✓	
[14]	Analysis of complex networks	✓		
[15]	Analysis of complex networks	✓		
[16]	Analysis of complex networks	✓		
[17]	Analysis of complex networks	✓		
[18]	Analysis of complex networks	✓		
[19]	Analysis of complex networks	✓		✓

**Table 2:** Network attributes.

Network	Nodes number	Edges number	Average weighted degree	Average clustering coefficient
*G*	197	2373	40.660	0.763

**Table 3:** List of softwares.

Software	Version
Python	3.9
NetworkX	2.8
Gephi	0.10

**Table 4:** Comparison results between MPR and CC.

Community	MPR (1, 2, 3)	CC (1, 2, 3)
Lung meridian	LU7, LU5, LU11	LU7, LU5, LU11
Large intestine meridian	LI4, LI11, LI15	LI4, LI11, LI15
Stomach meridian	ST36, ST25, ST40	ST36, ST25, ST40
Spleen meridian	SP6, SP9, SP10	SP6, SP9, SP10
Heart meridian	HT7, HT5, HT3	HT7, HT5, HT3
Small intestine meridian	**SI3, SI1, SI18**	**SI3, SI1, SI11**
Bladder meridian	**BL23, BL13, BL40**	**BL23, BL13, BL32**
Kidney meridian	KI3, KI1, KI6	KI3, KI1, KI6
Pericardium meridian	PC6, PC5, PC8	PC6, PC5, PC8
Triple burner meridian	TE5, TE17, TE14	TE5, TE17, TE14
Gallbladder meridian	GB20, GB34, GB30	GB20, GB34, GB30
Liver meridian	**LR3, LR2, LR14**	**LR3, LR14, LR2**
Governing vessel	**GV20, GV14, GV15**	**GV20, GV14, GV27**
Conception vessel	CV6, CV4, CV12	CV6, CV4, CV12
Extra points (head)	EX-HX5, EX-HX4, EX-HX12	EX-HX5, EX-HX4, EX-HX12
Extra points (dorsum)	EX-B1, EX-B2, EX-B9	EX-B1, EX-B2, EX-B9
Extra points (upper limbs)	**EX-UE9, EX-UE12, EX-UE10**	**EX-UE12, EX-UE9, EX-UE10**
Extra points (chest and abdomen)	EX-CA1	EX-CA1
Extra points (lower limbs)	EX-LE6	EX-LE6

The two algorithms in Table 4 provide the top 3 acupuncture points for each of the 19 regions. The locations highlighted in bold are used to indicate the regions where the two algorithms yield divergent results.

**Table 5:** Comparison results of the network efficiency.

Algorithm	MPR	Key node	CLD	CC	BC	EC
Efficiency	0.0008	2.4721	2.2307	2.6306	2.4249	2.4875

**Table 6:** Comparison results of the accuracy.

Algorithm	MPR (%)	Key node (%)	CLD (%)	CC (%)	BC (%)	EC (%)
Accuracy	63.3602	49.1914	42.1004	50.9391	52.6594	43.1007

## Data Availability

The data included in the paper to support the results of this research are described in detail in Section 2.1 for the data format and data sources. The data supporting this manuscript are available from the corresponding authors upon request to.
